# The Field of Disease: A Book of Preventive Medicine

**Published:** 1884-11

**Authors:** 


THE FIELD OF DISEASE: A BOOK OF PREVENTIVE MEDICINE. *By* BENJAMIN WARD RICHARDSON, M.D., LL.D., F.R.S. *8 vo.,*

*cloth, pp. 737. Philadelphia:* HENRY E. LEA'S SON & CO. 1884. *Chicago:* JANSEN, MCCLURG & CO.

As stated in the notice of the American publisher, this is the only work on preventive medicine accessible in the English language, and it is wisely written for, and addressed to, the public; for, as long as no provision exists for the reward of the prevention of any disease, although it may be now understood that the prevention of disease belongs to the function of the physician—still, the public finds it necessary to protect itself, and will find it necessary also to pay the services of its protectors even when they happen to be physicians.

No doubt the tendency on the part of the people in some sections of this country to employ family physicians by the year, gives the profession some facility for preventing a number of diseases by timely services; but this wise custom seems to decrease and to give place to the indiscriminate use of any medical man of any school, as emergency suggests. The role of preventive physician falls naturally to Boards of Health.

It is interesting to learn in the first chapter that there are: "*One thousand one hundred and forty-six* variations from health, making up the conditions and the phenomena of disease to which the human family, at this period of its civilization, may be subjected." But most of these are local affections; and of the 58 general diseases, 33 include the contagious and infectious, and 25 the great constitutional diseases.

The author's language, with a view to elucidate the text, contains many vulgarized scientific expressions, of which the following is an example: "If the body be exposed to agents which modify the vascular tension so that through the vessels the blood shall pass with undue friction, there may be an increase of fever which might be called *frictional fever.*" Whether this frictional fever will come into favor amongst physicians, and whether the "vascular tension" will be readily understood by the popular mind, remains to be seen.

The descriptions and definitions of the General Diseases are admirable for their terseness and clearness, and will be well understood by even illiterate readers.

The local diseases, on account of their variety, and for lack of a better classification than that commonly accepted, are not so satisfactorily described.

Medical readers will be surprised when looking at the paragraph on "*Membranous Croup*" to read that the disease "is not contagious," and that "it is considered as a local disease."

Of pneumonia, the author says that, "Essentially the disease is one, but various writers upon it speak of it as of different varieties." He omits to mention Croupous or Lobar Pneumonia, and says that the disease "is not contagious." Doubtless Dr. Richardson ought to read the article on Croupous Pneumonia, by Juergensen, in Ziemssen's Clyclopœdia, and many valuable reports on the subject by American writers.

Consumption is also treated of as a local affection ! ! Neurasthenia, of course, is not to be found in a work by an Englishman.

The Nomenclature of Mental (?) Diseases is restricted to Hypochondriasis, Mania, Melancholia, Dementia, Imbecility, and Idiocy, which occupy a little more than two pages.

Trichinosis is treated of as a local affection of the muscular system.

The third part of Book I. treats of Diseases from Natural Accidents, and contains much literature on medical topics rarely treated in ordinary works on Practice or Surgery.

Acquired Diseases from Inorganic and Organic Poisons form one of the most instructive and valuable parts.

Weather and Season as Causes of Disease have not been omitted, nor a discussion of Zymotic Causes; but the subject of cadaveric alkaloids as a factor in the Germ Theory is too recent a discovery to deserve attention in these pages.

Part the Second of Book III., which ought to have formed the bulk of this volume, being "A Practical Summary of Preventions of Disease," occupies the sixty-eight pages preceding the index. A separate publication of these few pages would confer a service on the profession in general, and on medical students in particular, while it might also suffice for the wants of the laity, few of whom, at any rate, can intelligently read the rest of the volume.

This latter part can never be too highly extolled, and would surely redeem faults of other portions if it was as extensive as it is valuable. To sum up, this being the only work treating of Preventive Medicine, should have an extensive sale amongst all educated classess, and its unavoidable faults should be overlooked in view of the crude state of our pathological learning as taught to-day. However, a few years of medical progress, stimulated by our lately established boards of health, will probably bring about, even during this generation, the means of providing far better knowledge of disease for the people at large, if not better means of prevention than found in this work. H. D. V.

